# Optimization of Supercritical Fluid Extraction of Oil from the Fruit of *Gardenia jasminoides* and Its Antidepressant Activity

**DOI:** 10.3390/molecules191219350

**Published:** 2014-11-25

**Authors:** Weiwei Tao, Hailou Zhang, Wenda Xue, Li Ren, Baomei Xia, Xin Zhou, Haoxin Wu, Jinao Duan, Gang Chen

**Affiliations:** 1 Center for Translational Systems Biology and Neuroscience, School of Basic Biomedical Science, Nanjing University of Chinese Medicine, Nanjing 210023, China; E-Mails: tw845@163.com (W.T.); hailouzhang@163.com (H.Z.); xuewenda@njutcm.edu.cn (W.X.); nicolight@163.com (L.R.); babaysummer@163.com (B.X.); aeon401@126.com (X.Z.); wuhaoxin@sohu.com (H.W.); 2 Laboratory of Integrative Biomedicine of Brain Diseases, School of Basic Biomedical Science, Nanjing University of Chinese Medicine, Nanjing 210023, China; 3 National and Local Collaborative Engineering Center of Chinese Medicinal Resources Industrialization and Formulae Innovative Medicine, Nanjing University of Chinese Medicine, Nanjing 210023, China; E-Mail: jinaoduan1956@126.com

**Keywords:** supercritical fluid extraction, response surface methodology, oil, *Gardenia jasminoides*, antidepressant effect

## Abstract

A response surface methodology was applied to optimize the variables affecting the supercritical fluid carbon dioxide extraction of oil from the fruit of *Gardenia* *jasminoides* using the Box–Behnken design. The optimum extraction parameters were an extraction temperature of 49.94 °C, an extraction pressure of 29.89 MPa and an extraction time of 93.82 min. Through a GC/MS analysis, we revealed 16 major components of the oil extract, which showed potent antidepressant effects in both of two behavior despair models in mice: tail suspension test and forced swimming test. Our results suggest that the oil extract of *Gardenia jasminoides* prepared using the supercritical fluid carbon dioxide extraction may contain effective constituents to be used for depression therapy.

## 1. Introduction

The dried ripe fruit of *Gardenia jasminoides* Ellis. has been used to extract natural food colorants and as an important oriental herbal medicine, which has been recorded as Fructus Gardeniae (FG) in the Chinese Pharmacopoeia and commonly used for the treatment of depression, anxiety, insomnia, psychosis and other mental disorders in Chinese traditional medicine [1,2]. The pharmacological actions of FG, such as protection against oxidative damage, anti-inflammatory activity, melanogenesis inhibitory activity and hepatoprotective activity, have been well characterized previously [3,4,5,6]. 

Previous studies have indicated that the effective chemical constitutes of FG extracts show a distribution of extracts in the range from mid-high to high polarity (e.g., aqueous extract, aqueous solution processed by using 95%–70% of ethanol) [7,8,9]. In addition, the water-soluble ingredients, such as iridoid glycosides and crocins, possess potentially notable antidepressant activity [1,10]. To search for novel active compounds responsible for antidepressant effects, the present study performs a phytochemical screening to obtain the non-polar oil from Fructus Gardeniae (OFG) and to evaluate its antidepressant activity.

Supercritical fluid extraction (SFE) is known to be a fast and efficient method for the extraction of non-polar compounds from plant matrices. CO_2_ extracts are generally recognized as safe for use in food and pharmaceutical production. It is an odorless, inexpensive, inert and environment-friendly solvent. The SF-CO_2_ technology has been applied in plant oil extraction from a large number of materials in pharmaceutical and food processing with a high potential for future applications [11,12].

However, to the best of our knowledge, the effect of SF-CO_2_ parameters on the OFG yield and the optimum operation conditions for OFG remain poorly investigated. For a possible industrial application, the optimization and assessment of the extraction process with mathematical modeling seem to be essential. In classical methods, the process parameters are optimized by conducting experiments concentrating on one factor at a time. This method is time consuming and troublesome and ignores the interaction effects of the parameters. Compared to the classical methods, the response surface methodology (RSM) is more efficient, requires fewer data and provides the interaction effects of the response besides the factor effects [13,14].

In the present study, SF-CO_2_ of oil from Fructus Gardeniae was studied. The effects of the main operating parameters, namely extraction temperature, extraction pressure and extraction time, on the extraction yields of OFG were investigated. The chemical composition of OFG was analyzed by GC-MS. Furthermore, the antidepressant activity of OFG was determined by means of a tail suspension test (TST) and a forced swimming test (FST), which have been used as primary screening tests for antidepressant drugs [15].

## 2. Results and Discussion

### 2.1. Optimization of the Extraction of OFG from FG Using RSM

The conditions for supercritical CO_2_ oil extraction from FG were optimized using different parameters in combination with the Box–Behnken design (BBD) (3^3^ factorial). The response values (oil yield) for different experimental combinations are given in Table 1. It can be seen from Table 1 that there is considerable variation in oil yield depending on the extraction conditions. The regression coefficients of the quadratic, linear and interaction terms of the model were calculated using the least squares technique and are presented in Table 2. It was evident that all quadratic parameters were significant (*p* < 0.01 or *p* < 0.05). All interaction parameters were insignificant (*p* > 0.05). By employing multiple regression analysis on data from the survey samples, the predicted response Y for OGF yield can be obtained by the following second-order polynomial equation:

Y = 11.41 + 0.31A + 0.94B + 0.44C − 0.20AB − 0.017AC − 0.04BC − 0.82A^2^ − 0.30B^2^ − 0.92C^2^, where A, B and C are in terms of coded factors of the test variables, extraction temperature, extraction pressure and extraction time, respectively.

**Table 1:** The response surface analysis programs and results of the oil yields (*n* = 3).

Standard	Run	A	B	C	Oil recovery (%)
12	1	45	30.0	120	11.30
4	2	60	30.0	90	11.39
11	3	45	15.0	120	9.57
13	4	45	22.5	90	11.48
2	5	60	15.0	90	9.83
10	6	45	30.0	60	10.89
14	7	45	22.5	90	11.37
8	8	60	22.5	120	10.59
9	9	45	15.0	60	9.00
5	10	30	22.5	60	8.72
3	11	30	30.0	90	11.16
6	12	60	22.5	60	9.36
16	13	45	22.5	90	11.31
17	14	45	22.5	90	11.41
7	15	30	22.5	120	10.02
15	16	45	22.5	90	11.45
1	17	30	15.0	90	8.79

**Table 2:** Analysis of variance.

Source	Sum of Squares	Degree of Freedom	Mean Square	*F*-Value	*p*-value Prob > *F*
Model	16.96	9	1.88	40.31	<0.0001
A-temperature	0.77	1	0.77	16.44	0.0048
B-pressure	7.13	1	7.13	152.39	<0.0001
C-time	1.54	1	1.54	32.94	0.0007
AB	0.16	1	0.16	3.51	0.1032
AC	1.225 × 10^−3^	1	1.225 × 10^−3^	0.026	0.8760
BC	6.400 × 10^−3^	1	6.400 × 10^−3^	0.14	0.7223
A^2^	2.8	1	2.8	59.89	0.0001
B^2^	0.37	1	0.37	8.00	0.0255
C^2^	3.55	1	3.55	75.89	<0.0001
-	*R*^2^ = 0.981	-	adjusted *R*^2^ = 0.957	-	-

The analysis of variance (ANOVA) for the experimental results of the BBD are also shown in Table 2. The high model *F*-value (40.31) and the very low *p*-value (*p* < 0.0001) implied that the model was valid. The value of *R*^2^ (0.981) revealed that the experimental data were in good agreement with the predicted values of the yield of seed oil. The value of adjusted *R*^2^ (0.957) suggested that the total variation of 95.7% for the yield of seed oil was attributed to the independent variables, and only about 4.3% of the total variation could not be explained by the model. In summary, the BBD showed that the polynomial regression model matched the experimental results well. Therefore, this model can be used to navigate within the range of the design space.

**Figure 1:**
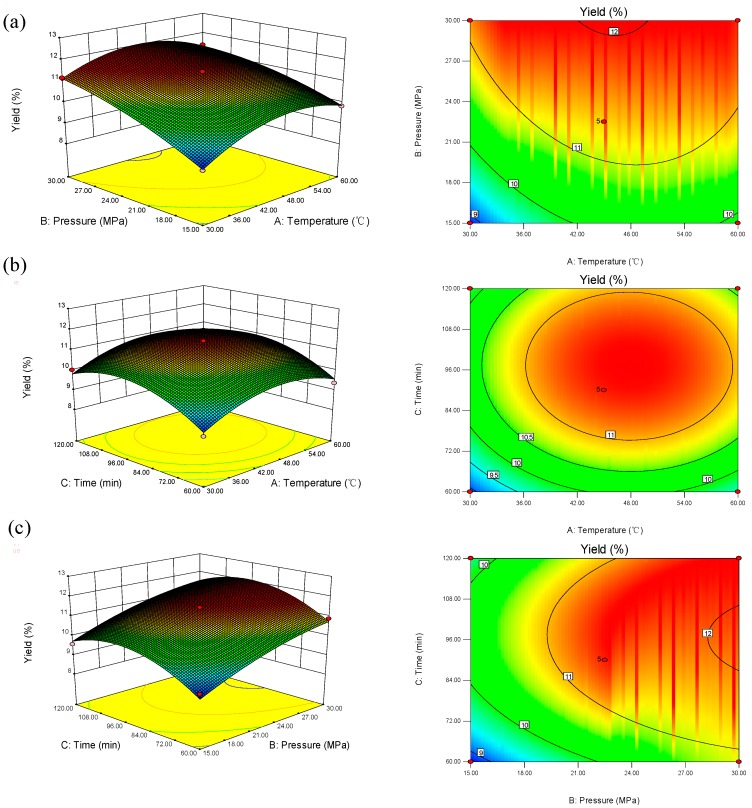
The 3D response surface and 2D contour plots of the oil recoveries affected by extraction pressure, extraction temperature and extraction time: (**a**) the effect of pressure and temperature on the oil yield at an extraction time of 90 min; (**b**) the effect of time and temperature on the oil yield at an extraction pressure of 22.5 MPa; (**c**) the effect of pressure and time on the oil yield at an extraction temperature of 45 °C.

The 3D response surface and 2D contour plots in Figure 1 provided a means of visualizing the relationship between the responses and experimental levels of each variable and the type of interactions between the two test variables. Figure 1a shows the effect of the extraction pressure and temperature on the oil yield at a fixed extraction time of 90 min. With a definite extraction temperature, pressure had a positive linear effect on the oil yield: the oil yield increased significantly with the extraction pressure (Figure 1a), most likely because of the increase in the CO_2_ solvent density, resulting in an improvement in oil solubility. However, the extraction temperature showed different results compared to the extraction pressure. The positive effect of temperature has been explained in terms of its ability to alter the physical properties of the sample matrix, making it easier for the CO_2_ to penetrate. Oil yields increased with the extraction temperature and reached a maximum value, followed by a decline with its further increase (Figure 1a). That was probably because of the fact that the density of the supercritical fluid decreased at a higher temperature. As shown in Figure 1b, with a fixed extraction pressure of 22.5 MPa, when the temperature was between 45 °C and 51 °C, an extraction time 90 and 102 min, oil yields reached the top; after this point, with time and temperature increasing, oil yields showed a downward trend (Figure 1b). Figure 1c shows the effect of the extraction pressure and time on the oil yield at a fixed extraction temperature of 45 °C. At a given extraction time, the oil yield increased rapidly with the extraction pressure, likely because the increase in the pressure led to the increase in the CO_2 _density, which subsequently induced the improvement in solvent power.

Since the extraction of other impurities continued to accumulate, the optimum values were found by solving the regression equation and response surface analytically. The predicted oil yield was 12.03% and lay in the following ranges of the examined variables: extraction temperature 30–60 °C, extraction pressure 15–30 MPa and extraction time 60–120 min. The optimum values of the test variables were extraction temperature, 49.94 °C, extraction pressure, 29.89 MPa, and extraction time, 93.82 min.

The trial experiments were conducted under optimized conditions. Taking convenience into account, the optimum experimental parameters were determined as follows: extraction temperature, 50 °C, extraction pressure, 30 MPa, and extraction time, 94 min. To compare the predicted results (12.03%) with experimental values, rechecking was performed using deduced optimal conditions. The mean value of 12.11% (*n* = 3), obtained from experiments, showed the validity of this RSM model, because the differences between 12.03% and 12.11% (*n* = 3) were not significant (*p* > 0.05). The strong correlation between experimental and predicted results confirmed that the response model was accurate and adequate to reflect the expected optimization of the oil extraction process.

### 2.2. Chemical Compositions of OFG

The oil yield of SF-CO_2_ from FG was 8%–12% (*w/w*) according to the raw material. A total of 16 main constituents of OFG were identified, as shown in Table 3. Relative content was calculated by integrated peak area in the Agilent data analysis program. Most of the constituents in the OFG extract can be classified into different chemotypes, including monoterpenes (25.15%), sesquiterpenes (1.98%), fatty acids (29.0%), long-chain alkenes (23.41%) and others. The major compounds in OFG were quantified as being myristic acid (15.30%), pentadecane (15.14%), palmitic acid (13.70%), 2,6,6-trimethyl-1,3-cyclohexadiene-1-carboxaldehyde (11.72%) and 2,2,6-trimethylcyclohexanone (9.65%), based on the results obtained from GC/MS.

**Table 3:** Chemical compositions of oil from Fructus Gardeniae.

No	Component	Relative Content	Molecular Formula	Molecular Weight	Degree of Similarity (%)
1	2-Heptenal	0.72	C_7_H_12_O	112	98
2	2-Pentylfuran	3.37	C_9_H_14_O	138	97
3	Caproic acid	0.24	C_6_H_12_O_2_	116	99
4	2,2,6-Trimethylcyclohexanone	9.65	C_9_H_16_O	140	98
5	2,5-Dimethylbenzaldehyde	3.23	C_9_H_10_O	134	96
6	Linalool	3.96	C_10_H_18_O	154	98
7	2,6,6-Trimethyl-1,3-Cyclohexadiene-1-Carboxaldehyde	11.72	C_10_H_14_O	150	98
8	Eucarvone	2.17	C_10_H_14_O	150	98
9	2,4-Decadienal	7.30	C_10_H_16_O	152	99
10	β-Elemene	1.22	C_15_H_2_	204	95
11	α-Guaiene	0.76	C_15_H_24_	204	96
12	Pentadecane	15.14	C_15_H_32_	212	97
13	Heptadecane	6.16	C_17_H_36_	240	98
14	2-Methyl-Heptadecane	2.11	C_18_H_38_	254	97
15	Myristic acid	15.30	C_14_H_28_O_2_	228	96
16	Palmitic acid	13.70	C_16_H_32_O_2_	256	98

### 2.3. Antidepressant Activity Evaluation

#### 2.3.1. Tail Suspension Test

**Figure 2:**
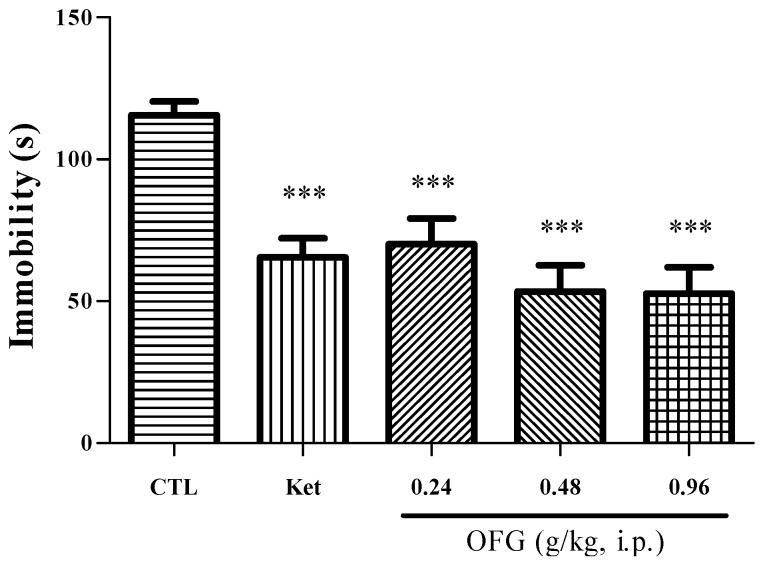
Antidepressant effect of oil from Fructus Gardeniae (OFG) and ketamine in the tail suspension test. The values are the mean ± SEM (*n* = 12) for each group. One-way analysis of variance (ANOVA); *** *p* < 0.001 compared to the control group (CTL).

As shown in Figure 2, at 24 h after administration of ketamine (30 mg/kg) or different doses of OFG (0.24 g/kg, 0.48 g/kg, 0.96 g/kg) in mice, there was a significant effect of treatment (ANOVA, *F* = 10.64, *p* < 0.001). Ketamine (30 mg/kg, *p* < 0.001) significantly reduced the immobility time as compared with the control group that received saline. All doses of OFG significantly reduced the immobility time (*p* < 0.001, *post hoc*).

#### 2.3.2. Forced Swimming Test

**Figure 3:**
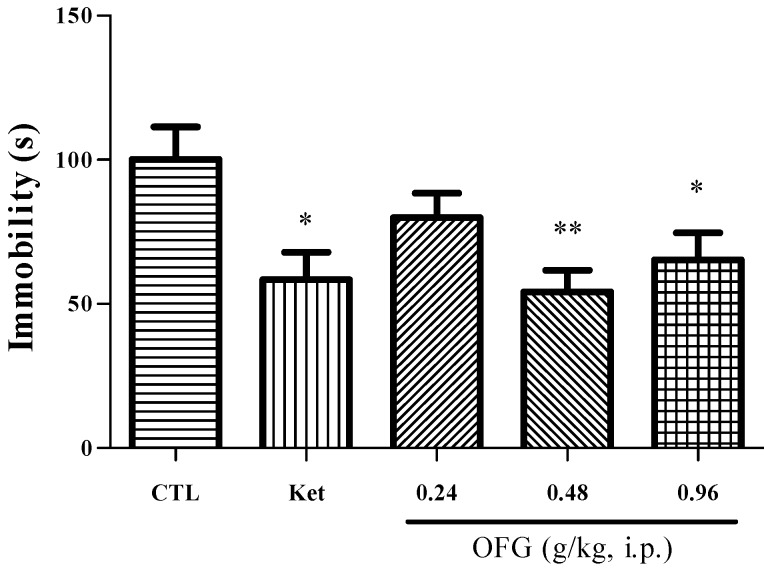
Immobility time of OFG and ketamine in the forced swimming test. The values are the mean ± SEM (*n* = 12) for each group. One-way analysis of variance (ANOVA); ** *p* < 0.01 and * *p* < 0.05 compared to the control group (CTL).

The behavioral effects produced in the FST test at 24 h post drug administration are presented in Figure 3. No significant reduction in immobility time was observed in the mice treated with the dose of 0.24 g/kg OFG. The other OFG groups and ketamine showed a significant reduction (*p* < 0.05, *post hoc*).

The data presented have demonstrated that OFG significantly reduced the immobility time in the TST and FST, indicating significant antidepressant effects in the two behavior despair models that are most widely used. These results suggest that the non-polar fraction contributed remarkably to the antidepressant effect of FG.

## 3. Experimental Section

### 3.1. Animals

Male outbred Kunming were used in the present study. Mice aged 6–8 weeks old (18–22 g) were habituated to animal facilities for 1 week before behavioral testing. Mice were kept on a 12-h dark-light cycle, with free access to water and food. For all behavioral testing, mice were weight matched. All animal procedures were in accordance with the Guide for the Care and Use of Laboratory Animals in China and were approved by the Institutional Animal Care and Use Committee at Nanjing University of Chinese Medicine. The experimenters were blinded to the assignments of the mice.

### 3.2. Materials

The plant, *Gardenia jasminoides* Ellis., was grown in the planting base of Fengcheng, Jiangxi province (China). Fructus Gardeniae were ground into powder in a cyclone mill and passed through a 40 mesh sieve (particle size, 0.45 mm) for supercritical fluid extraction. All solvents used in the analysis were of analytical grade. OFG was dispersed in Tween 80 solution (0.5%, *w/v* in saline). Vehicle solvent (0.1%, *w/v* Tween 80 in saline) served as the negative control. The solutions of the SF-CO_2_ extraction and vehicle were administered to the mice via intragastric administration. Ketamine HCl (Gutian Pharmaceuticals, Fujian, China), dissolved in saline, was administered intraperitoneally.

### 3.3. SFE-CO2 Extraction

SFE was carried out using the Model HA121-50-01 extraction system (Hua’an Supercritical Fluid Extraction Corp., Nantong, China) equipped with a 1000-cm^3^ extraction vessel in which 300 g of FG powder were loaded for each experimental run. The supercritical fluid flow rate of CO_2_ was 25 kg·h^−1^. During the extraction process, the extraction temperature, extraction pressure and extraction time were controlled by regulating the adjusting valves on the front panel. When the scheduled time was achieved, supercritical CO_2_ was depressurized, and the oil was collected in a collection vial. The oil obtained under the optimal conditions was used for the following tests. The amount of extracted oil was determined gravimetrically after collection, and then, the extraction yield was expressed as the percent ratio of the mass of extracted oil to the mass of raw material loaded in the extraction tube, as follows:

Extraction yield of FG oil (%) = (mass of extracted oil/mass of dried material) × 100%
(1)

### 3.4. Experimental Design and Statistical Analysis

Three factors (extracting temperature, extracting pressure and extracting time) were chosen based on preliminary experiments for further optimization by employing a three-level, three-variable Box–Behnken design (BBD) from RSM. Codes and levels of independent variables of concentration temperature, pressure and time in the RSM design are given in Table 4. The whole design consisted of seventeen experimental points carried out in random order. Five replicates at the center of the design were used for estimating the pure error sum of squares (Table 1). Regression analysis was performed for the experimental data and was fitted into an empirical second-order polynomial model, as shown in the following equation:

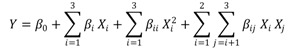
(2)

Here, the average oil yield (%) was taken as the response, Y, β_0_, β_i_, β_ii_ and β_ij_ are the regression coefficients of the variables for intercept, linear, quadratic and interaction terms, respectively, and X_i_ and X_j_ are independent variables (i ≠ j). The coefficients of the second-order polynomial model and the responses obtained from each set of experimental designs were subjected to multiple nonlinear regressions using Design-Expert software (Design-Expert. V8.0.6, Stat-Ease, Minneapolis, MN, USA). The quality of the fitted model was expressed by the determined coefficient (*R*^2^), and its statistical significance was checked by an *F*-test.

**Table 4:** Codes and levels of independent variables of concentration temperature, pressure and time in the response surface methodology (RSM) design.

Symbol	Independent Variable	Coded Levels		
		−1	0	1
X_1_	Temperature (°C)	30	45	60
X_2_	Pressure (MPa)	15	22.5	30
X_3_	Time (min)	60	90	120

### 3.5. Gas Chromatography-Mass Spectrometry Analysis

GC-MS analysis was performed using an Agilent HP4890D gas chromatography/mass spectrometer (Agilent, Santa Clara, CA, USA), equipped with an HP-5capillary column (30 m × 0.25 mm × 0.25 μm). The column temperature was initially at 40 °C (held for 4 min), then increased to 120 °C at 10 °C/min and finally, increased to 260 °C at 5 °C/min, held for 20 min. The mass spectrometer was operated in positive ion mode with ionization energy of 70 eV. Helium was used as the carrier gas at a flow rate of 1.2 mL/min. The identification of oil components was based on matching their recorded retention indices and mass spectra with those in the NIST (National Institute of Standards and Technology, Gaithersburg, MD, USA) library data provided by the GC/MS software.

### 3.6. Antidepressant Activity Evaluation

#### 3.6.1. Tail Suspension Test (TST)

The procedure for TST was reported as previously described [16]. Mice were assessed in the TST, which was performed with a computerized device allowing four animals to be tested at one time. In a chamber that was both acoustically and visually isolated, an individual mouse was suspended 50 cm above the floor by adhesive tape placed approximately 1 cm from the tip of the tail. The sessions of the animals were videotaped. Data were calculated for the total duration of immobility in the final 4 min of the 6-min test. Immobility was scored as a failure to make any struggling movements, attempts to catch the adhesive tape or body torsions or jerks.

#### 3.6.2. Forced Swim Test (FST)

The FST was carried out in mice, individually forced to swim in an open cylindrical container (diameter 10 cm, height 25 cm), containing 15 cm of water at 25 ± 1 °C. The sessions of the animals were videotaped. Data were calculated for the total duration of immobility in the final 4 min of the 6-min test. Each mouse was considered to be immobile when it failed to struggle and remained floating motionless in the water, making only those movements necessary to keep the nose above water. A decrease in the duration of immobility during the FST was taken as a measure of antidepressant activity. Statistics for TST and FST were made using one-way ANOVA, followed by the Newman–Keuls multiple range test. All data are presented as the mean ± SEM, and statistical significance was accepted at the 5% level, unless otherwise indicated.

## 4. Conclusions

The extraction yield of non-polar oil via SF-CO_2_ was optimized by RSM by using the extraction temperature, pressure and time as independent variables. The experiment results showed that the optimal conditions for the production of OFG were the following: extraction temperature, 49.94 °C, extraction pressure, 29.89 MPa, and extraction time, 93.82 min. Our study reveals the chemical compositions of the non-polar constituents from FG and demonstrates that the extract fraction has a potential for the development of novel antidepressant food supplements and medicines.
